# The Institutional Library

**Published:** 1921-02-26

**Authors:** 


### THE INSTITUTIONAL LIBRARY.

**Hypnotism and Treatment by Suggestion.** By Albert E. Davis, F.R.C.S. (Edin.), L.R.C.P. Third and Enlarged Edition. (Liverpool : The Liverpool Booksellers Co., Ltd. London : Simpkin, Marshall & Co., Ltd. Pp. 196. Price 5s. net.)

The inexact sciences form a happy hunting-ground for the theorists wherein they may disport themselves and indulge their propensities among a matter of words. One theory being as good—or as bad—as another, there is no need for humility in advocating the chosen one. Hence we are bowed down beneath a fearsome weight of " psychological " literature. But Dr. Davis is not one of those who chastises us with scorpions. There is a commendable brevity about his book which makes it easy to understand why the demand for it has continued, and his lucidity of expression is grateful and comforting to anyone who has suffered a blight from neologisms and periphrases. There is, of course, a danger even in being too succinct, and this danger he has not quite avoided. It leads to the suspicion that possibly in some cases diagnosis was not quite exact, and that, therefore, the " cures " were not so remarkable as they might have been. For example, he speaks of cure in a case of early locomotor ataxia by suggestive therapeutics. A little scepticism may be permitted. Altogether, Dr. Davis is a confirmed optimist in so far as his methods ef treatment are concerned. Perhaps this is just as well, and it may have accounted for a number of his successful results by enabling him to inspire confidence in his patients of a happy issue from their troubles. It seems to lead him astray at times, and to make him disparage unduly other methods than his own. The use of drugs, it may be admitted, is still overdone, especially hypnotic drugs. But when Dr. Davis says that " drugs cannot cure insomnia ; on the contrary, they increase it, making the sufferer's last condition worse than the first," one can only wonder whether he has had much experience of cases of acute insanity, and, if so, has he been able to cure their insomnia by " magic numbers and persuasive sound " ? Apart from certain blemishes, however, Dr. Davis has written a most interesting book. There is much sound common sense in it. He utters a very necessary warning against the improper use of hypnotism by charlatans and by those who employ it as a means of amusing the public, and his plea for the re-education of the paralysed is well worthy of attention. There is a brief but comprehensive sketch of the history of treatment by suggestion. Altogether a most readable and—with certain limitations—most instructive book.

**Genito-Urinary Surgery and Venereal Diseases.** By E. Martin, M.D. ; B. A. Thomas, M.D. ; and S. W. Moorhead, M.D. Twelfth Edition. (Lippincott Co. 1920. Pp. 928. Price 35s. net.)

This text-book, originally written by White and Martin, has been drastically revised and rewritten since pre-war days. It represents the very best class of American surgical monograph, well balanced, well written, sensible, up to date. The illustrations are a strong point : those in colour are quite good, but are equalled by a good many other books ; those in ordinary photo-printing are of quite unusual excellence and deserve a special note of commendation. It is interesting to observe how very closely the best American and the best British practice in this specialty agree ; in the vast development of genito-urinary surgery of the last quarter of a century, surgical opinion is each nation has marched side by side with that in the other, to all intents.

In a detailed examination there is little found which need qualify this expression of opinion. There are, however, other methods of nephropexy besides that described in this book ; and some British operators hold that they are vast improvements on the older operation here described. Mr. Kidd's view as to the causation of subacute epididymitis by endogenous infection with *Bacillus coli*, and of the mistaken diagnosis of tuberculosis to which it sometimes gives rise, has either not reached or not been accepted by the authors of this book. There can be little doubt that so long as this text-book is edited as carefully, and published as admirably, as this is, it will continue the steady process of edition followed by edition.

**A Synopsis of Medicine.** By H. L. Tidy, M.D., F.R.C.P. (Bristol : John Wright & Sons. 1920. Pp. 952. Price 26s. net.)

Attempts to compress the really essential facts of the now vast and ever-expanding science of medicine into a reasonable compass demand a very nice judgment as well as an encyclopædic knowledge. Naturally there is a strong family resemblance between the products of men who fulfil these conditions, and there is therefore no harm in saying that Dr. Tidy's attempt is reminiscent of some of his predecessors who have been most successful ; for this new condensation is exceedingly well done. To turn up one's nose at books of this type as " examination cram-books " is unfair and ungenerous. Doubtless they have a definite value to those on the eve of examination ordeals ; but they have also a very real and distinct value to the busy practitioner who wishes to consult an authority, both up-to-date and not too discursive, on some point in connection with a case of disease.

In such a comprehensive survey it might be easy to find small points of disagreement, were one to spend time searching for them ; for instance, on the value which Dr. Tidy appears to attach to vaccine treatment in erysipelas. It is more useful to acknowledge the wide purview and sound common-sense which pervades the work as a whole. Occasional omissions, very probably intentional, exist ; for instance, the classification of human blood (from the transfusion point of view) is to all intents omitted. The handling of type founts of many kinds, to secure the illumination and elucidation of the text, has been extremely well done. Dr. Tidy's task must have involved enormous labour, but it has been well worth while.

**Handbook of Physiology.** By W. D. Halliburton, M.D., LL.D., F.R.C.P., F.R.S., Professor of Physiology, King's College, London. (London : John Murray, Albemarle Street. 1920. 18s. net.)

The fifteenth edition of this well-known text-book differs in no great essentials from its predecessor. Changes are of a minor kind, and those which have been made are only necessary to make its modernisation even more proper. We can only repeat our favourable criticism of the work.

**Catechism Series: "Surgical Anatomy"** Part II. By C. R. Whittaker. (Edinburgh : E. & S. Livingstone. Second Edition. Price 1s. 9d.)

This volume on the abdomen and thorax maintains the high standard of this excellent series. The student is provided with a method of revising his work which, though it may not be academic, at least possesses the merit of efficiency.

